# Importance of Translational Research for Targeting Fibroblast Growth Factor Receptor Signaling in Cancer

**DOI:** 10.3390/cells8101191

**Published:** 2019-10-02

**Authors:** Klaus Holzmann, Brigitte Marian

**Affiliations:** Medical University of Vienna, Comprehensive Cancer Center, Department of Medicine I, Division of Cancer Research, Borschkegasse 8a, 1090 Vienna, Austria

Fibroblast growth factors (FGFs) are a large family of protein ligands that exert a wide range of biological effects in many organs/tissues by activating receptors (FGFRs) of the tyrosine kinase superfamily [1,2]. They are crucial for embryonic development as well as for tissue maintenance and repair in the adult organism [3]. Based on these physiological functions it is not surprising that FGFR signaling is dysregulated in practically every malignancy that has been analyzed in this context [4]. The FGFR activation is common in different tumor types, but only <10% of all tumors sequenced carry FGFR aberrations, such as gene amplifications, mutations and rearrangements [5]. Most commonly affected (up to 32%) are specific tumor types such as urothelial, breast, endometrial and squamous cell lung cancer. The more frequent mechanism is the upregulation of FGFs to establish autocrine and paracrine loops [6,7,8]. This adds an additional layer of complexity, because the secreted factors also affect cells of the microenvironment while FGFs produced in the microenvironment may stimulate the cancer cells [9].

Efforts to target FGF signaling in tumors have been going on for about a decade and produced several mostly multi-target compounds that inhibit vascular endothelial growth factor and platelet-derived growth factor in addition to FGFRs. Several such inhibitors are already in clinical trials or used as cancer drugs [10,11]. With regard to the FGFR family, FGFRs1-3 are so closely related that small molecule inhibitors usually affect all 3 in a similar way. Only for FGFR4 with its distinctly different kinase domain, a specific inhibitor has been developed [10,12].

There is still much we do not know: the intricate signaling network underlying the impact of FGFs on the growth, survival and invasiveness of cancer cells and the interaction of FGF-signaling with healthy cells in a paracrine manner driving angiogenesis and metastasis need to be further elucidated to define therapeutic targets and predictive markers for cancer therapy. Since 2017 several excellent articles about general FGFR targeting in cancer have been published, e.g., [10,13]. However, a translational perspective of targeting FGFR signaling for specific cancer subtypes was currently the main topic of only a limited number of review articles, e.g., for squamous cell lung cancer [14], breast cancer [15], endometrial cancer [16], pancreatic cancer [17], prostate cancer [18], and focusing on FGFR4 signaling in hepatocarcinogenesis [19].

This Special Issue of *Cells* undertakes to cover translational research on FGFR signaling from basic science to clinical studies with strong emphasis on the improvement of knowledge for clinical application. Our call for this special issue entitled “Fibroblast Growth Factor Receptor (FGFR) Signaling Pathway in Tumor” resulted in a total of 15 published articles, including seven reviews.

This specific collection of seven review articles delineate expression and targeting options extending the current knowledge about the aforementioned cancer subtypes for glioblastoma [20], gastric cancer [21] and skin cancer [22] and provides updates about hepatocellular carcinoma and targeting FGFR4 signaling [23,24]. It includes structural information about FGFRs important for development of small molecule inhibitors [25] and offers information about the regulation of FGFRs especially by plasma membrane-embedded partner proteins that may act as coreceptors [26]. In hepatocellular carcinomas [23], but also in some other malignancies [24], upregulation of FGFR4 is coupled to secretion of FGF19 to form an autocrine loop and offers a promising therapeutic target— especially as FGFR4-specific targeting compounds have been developed and are already in clinical trials [24]. Dai et al. give a comprehensive overview of the development of FGFR inhibitors and their specificities in relation to their interaction with the FGFR kinase domains [25]. Czys reports in her review on melanomas that alterations in FGF-signaling are not driving the malignant process, but they do increase with tumor progression and contribute to more aggressive phenotypes and therapy resistance [22]. Consequently, targeting FGFRs is suggested for combination therapy [22]. Similar observations have been reported for other malignancies, such as colon cancer [27,28], mesothelioma [29], and lung cancer [30].

Of the reports on original data, two articles by Nanni et al. and Csanaky et al. contribute results on FGFR-dependent signaling and its biological impact on autophagy and differentiation in non-malignant in vitro cell models [31,32]. FGFR variant expression and subcellular localization are essential for the observed biological effects that could impact carcinogenesis. For example, the expression of mesenchymal FGFR variants, such as the IIIc alternative splicing variant in epithelial tumor cells, may increase FGFR signaling via paracrine FGF ligand effects [33]. Szybowska et al. analyzed the impact of FGFR2 mutations on downstream signaling and feed-back loops [34]. Santolla et al. address the issue of tumor cell–microenvironment cross-talk, as they report on interaction with the G-protein estrogen receptor upregulating FGF2 in cancer associated fibroblasts that in turn impacts on the FGFR1 expressing breast cancer cells [35].

More tumor type-specific aspects are taken up in four research articles. Celik-Selvi et al. studied members of the Sprouty protein family that are well-known to inhibit FGFR signaling but some show a tumor-promoting function in brain cancer [36]. Vlacic et al. report about the expression of FGFRs and their prognostic significance in a very rare malignancy—malignant pleural mesothelioma [37]—and Jomrich et al. have analyzed FGFs as prognostic markers in adenocarcinomas of the esophageal–gastric junction [38]. Sarcomas exhibit predominant FGFR1 expression that can be specifically blocked in vitro in human and canine cell models [39]. FGFR expression profiles and blocking capacity were identical and support future comparative research in both species. In this Special Issue, a preclinical study in vivo by Hanes et al. identified amplified FRS2 as the determinant of response to FGFR-inhibitors in high-grade metastatic dedifferentiated liposarcoma, thus paving the way for clinical trials with a pan-FGFR inhibitor that may be more potent to block FGFR signaling in this specific sarcoma subtype [40].

In conclusion, the data presented in this Special Issue extends our knowledge on targeting FGFR signaling for cancer therapy to new compounds/strategies and to new tumor types. They also demonstrate the need for further translational research to decipher the complex role of FGFR signaling for improved targeting in different cancer subtypes.
